# Optimal exogenous calcium alleviates the damage of Snow-melting agent to *Salix matsudana* seedlings

**DOI:** 10.3389/fpls.2022.928092

**Published:** 2022-09-28

**Authors:** Hui Li, Shenglan Huang, Chengshuai Ren, Xiaohang Weng, Songzhu Zhang, Liying Liu, Jiubo Pei

**Affiliations:** ^1^ College of Forestry, Shenyang Agricultural University, Shenyang, China; ^2^ Research Station of Liaohe-River Plain Forest Ecosystem, Chinese Forest Ecosystem Research Network (CFERN), Shenyang Agricultural University, Changtu, China; ^3^ College of Land and Environment, Shenyang Agricultural University, Shenyang, China

**Keywords:** exogenous calcium, *Salix matsudana*, NaCl stress, photosynthetic characteristics, stress resistance

## Abstract

As the main component of snowmelt agents, NaCl is widely used in northern winters and significantly impacts the expected growth of garden plants in north China. *Salix matsudana* is also faced with salt stress caused by snowmelt, which seriously affects its development as the main tree species in the northern landscape. However, how exogenous calcium alleviates salt stress in *Salix matsudana* is not yet clear. In this study, the indicators of growth indices, photosynthetic characteristics and stress resistance were measured by hydroponic assays in combination with three NaCl conditions (0, 50 and 200 mmol·L^-1^) and five calcium concentrations (0, 2.5, 5, 10 and 20 mmol·L^-1^). The study’s results indicated that the application of exogenous calcium remarkably promoted the growth of *Salix matsudana* seedlings under NaCl stress. When the exogenous calcium concentration was 10 mmol·L^-1^, the plant height and basal diameter of *Salix matsudana* seedlings increased significantly, and the biomass of all parts reached the maximum (*P<* 0.05). Exogenous calcium can substantially improve the photosynthesis of *Salix matsudana* seedlings under salt stress. The photosynthetic parameters, photosynthetic pigment content and photosynthetic product synthesis of *Salix matsudana* seedlings were significantly increased at an exogenous calcium concentration of 10 mmol·L^-1^, and the photosynthetic level of *Salix matsudana* seedlings reached the highest value. The chlorophyll fluorescence parameters (*F*_v_ */F*_m_, *F*_v_ */F*_0_) of *Salix matsudana* seedlings were significantly decreased under different concentrations of NaCl stress. The maximum photochemical efficiency (*F*_v_ */F*_m_) and potential photochemical efficiency (*F*_v_ */F*_0_) of *Salix matsudana* seedlings peaked when the exogenous calcium concentration was 10 mmol·L^-1^, which was significantly higher than that of the other treatments (*P<* 0.05). The water use efficiency of *Salix matsudana* was affected considerably by NaCl stress. The WUE and iWUE peak values of *Salix matsudana* were significantly higher than those of other calcium concentrations at 10 mmol·L^-1^ (*P<* 0.05). Exogenous calcium can increase the activities of CAT, SOD and POD enzymes in *Salix matsudana* seedlings under different NaCl concentrations. Under NaCl stress, adding exogenous calcium promoted the survival rate and growth of *Salix matsudana* seedlings. In conclusion, the optimum exogenous calcium concentration for *Salix matsudana* seedlings was 10 mmol·L^-1^. High or low concentrations of exogenous calcium did not achieve the best results in alleviating salt stress in *Salix matsudana*.

## 1 Introduction

Snowmelt agents with NaCl as the main component have caused environmental problems in northern China (Wang et al., 2017). Each year, approximately 75-90% of China’s melting snow salt enters green belts along roads, threatening street trees (Yan et al., 2017), such as willow or poplar. With deicing salts, the surrounding ecology can be compromised: seed germination is reduced (Haiyun et al., 2002), and ground and surface water can be contaminated (Blasius and Merritt, 2002; Godwin et al., 2003; Rivett et al., 2016). Chlorine salts accumulate and infiltrate into plant roots, severely affecting the growth of green tree species and damaging the cell membranes of roadside plants (Yu and Tang, 1998; Alshammary et al., 2004). Snowmelt exposes roadside plants to more severe salt damage, such as leaf browning, physiological drought, specific ion poisoning and disruption of normal metabolism (Dai et al., 2012; Fan et al., 2013; Lee et al., 2017), which affects plant growth and may even lead to plant death. This, in turn, may lead to long-term environmental problems such as rapid accumulation of soil salts, soil consolidation and soil salinization (Pedersen et al., 2000). Research has shown that when the salt content of the soil surface exceeds 0.6%, most plants, except for some salt-tolerant species, fail to grow normally or even die, specially cultivated plants (Tao et al., 2018). Therefore, reducing salt stress caused by snowmelt agents on plants should be considered.

Calcium, as one of the main elements required by plants, has an essential role in plant physiological processes (Yang et al., 2014). Calcium is a signalling molecule that promotes plant growth under salt stress and is involved in various developmental processes and water transport, photosynthesis and mineral nutrition, as well as in plant growth and developmental processes and the regulation of stress signals (Pathak et al., 2020). Ca^2+^ also maintains plant cell membranes’ relative structural and functional integrity (Tuna et al., 2007). It plays a crucial role in stabilizing the structure of the cell wall, controlling ion selection and transport, cell wall enzyme activity and ion exchange behaviour (Ashraf and Harris, 2004). Ca^2+^ protects plants from oxidative stress by enhancing the activity of antioxidant enzymes (Khan et al., 2009; Arshi et al., 2010; Cha-um et al., 2012) and counteracts osmotic stress imbalance by increasing transpiration by altering the transpiration rate and stomatal conductance of plants (Gilliham et al., 2011; Yang and Guo, 2017). Applying exogenous calcium can alleviate salt stress and mitigate the damage caused by salt stress (Yang et al., 2022). The addition of exogenous calcium can help salinity-induced damage to calcium influx, transport and supply in plants (Ding et al., 2010; Pathak et al., 2020). The chlorophyll content of salt-stressed plants increased after applying calcium treatment to inhibit the accumulation of H_2_O_2_, thereby avoiding oxidative damage in plants (Tahjib-Ul-Arif et al., 2018). In pot culture experiments with *Dioscorea rotundata* plants, calcium-mediated improvement in salt stress was found to increase catalase (CAT) and superoxide dismutase (SOD) activities (Jaleel et al., 2008). Salt stress is minimized when maize, tall fescue and reed canary grass seedlings are exposed to calcium (Maeda and Nakazawa, 2008). The addition of Ca^2+^ increased the relative water content of rice seedlings under salt stress without increasing excess proline, demonstrating the importance of Ca^2+^ in maintaining water balance in plants under salt stress (Khan et al., 2009; Arshi et al., 2010; Cha-um et al., 2012). Yang et al. (2010) observed the effects of calcium supplementation on tomato (*Lycopersicon esculentum*) plants at different times, demonstrating that the coexistence of salt and calcium in the growth medium is necessary to alleviate salt stress and that calcium has no ameliorating effect until salt stress occurs. Studies have shown that exogenous calcium can relieve salt stress in strawberries, tomatoes, rice and iris to varying degrees (Kaya et al., 2002; Tuna et al., 2007; Rahman et al., 2016; Liu et al., 2021). The increased net photosynthetic rate in rice (*Oryza sativa* L.) was attributed to exogenous calcium and increased synthesis of osmotic substances in the leaves. Therefore, oxidative damage in plants treated with simultaneous application of NaCl + Ca^2+^ was significantly less than in plants treated with NaCl alone (Roy et al., 2019).

*Salix matsudana*, a common tree species in northern urban landscaping, is usually used as a shade tree or street tree and has excellent resistance to pollution. *Salix matsudana* has good characteristics, such as waterlogging resistance, saline-alkali resistance and developed root systems. *Salix matsudana* is highly adaptable and can produce many high-quality, live seedlings in a short period through asexual reproduction, which has high economic and ornamental value. The expected growth of *Salix matsudana* has been seriously affected by the use of large amounts of snowmelt in northern winters. There is a lack of research on exogenous nutrients to alleviate salt stress in landscape plants. Therefore, we propose the following hypothesis: applying exogenous calcium can lessen the harm caused to *Salix matsudana* by using snowmelt in winter. The optimum calcium concentration could promote the growth, biomass, photosynthesis and stress resistance of *Salix matsudana* seedlings under salt stress. This study provides a theoretical basis for further research on the effect of calcium on the growth and development characteristics of *Salix matsudana* and for scientific application of Ca fertilizer to improve the quality of *Salix matsudana* growth in landscape gardening.

## 2 Materials and methods

### 2.1 Experimental design

This experiment was conducted in a greenhouse at Shenyang Agricultural University from April to July 2019. A hydroponic experiment was conducted to eliminate soil components’ interference with the *Salix matsudana* seedlings. One-year-old *Salix matsudana* seedlings were obtained directly from the field after they had been rejuvenated and pruned, then selected seedlings with consistent long momentum were transplanted into a polypropylene (PP) box with a size of 320 × 180 × 140 mm (length × width× height). A layer of sponge with three small holes was placed on the culture box. One *Salix matsudana* seedling was fixed in each hole. Seven L solution without Na^+^ and Ca^2+^ was added to each tube to ensure the seedlings’ roots were immersed in the solution. Those were treated with the combined solution with three levels of NaCl (0, 50 and 200 mmol·L^-1^) and five levels of calcium (0, 2.5, 5, 10 and 20 mmol·L^-1^ CaCl_2_), according to the most salt stress and calcium experiments (Paiva et al., 1998; Fuller et al., 2012). A total of fifteen treatments were included, each with three replicates. The pH of the solution was controlled at 5~6 by adding NaOH. Other compounds that provided a large number of elements were KNO_3_, MgSO_4_·7H_2_O, KH_2_PO_4_, EATA-Na_2_ and FeSO_4_·7H_2_O. Trace elements were provided by H_3_BO_3_, MnCl_2_·4H_2_O, H_2_MoO_4_·H_2_O, ZnSO_4_·7H_2_O and CuSO_4_·5H_2_O. Every two days, the solution was checked and supplemented to maintain the volume at 7 L. During seedling growth, each treatment was equipped with an air pump, which was continuously ventilated from 7:00 to 19:00 and ventilated for 1 hour every two hours after 19:00. Other management measures were carried out under the routine. Every week, all boxes were stochastically allocated to different positions to reduce the influences of potential environmental factors.

### 2.2 Determination of growth indices of *Salix matsudana* seedlings

#### 2.2.1 The basal diameter and height of *Salix matsudana* seedlings

In July 2019, the basal diameter and height of the *Salix matsudana* seedlings were measured before destructive harvesting. The basal stem was measured with a Vernier calliper to 0.01 mm, and the height of the plant was measured with a ruler to 0.10 cm.

#### 2.2.2 Biomass of *Salix matsudana* seedlings

During seedling harvesting, the non-destructive *Salix matsudana* seedlings were removed from the box for each treatment in July 2019. The roots, stems, leaves and lateral branches were sampled, washed with distilled and ultrapure water, and immediately dried with tissue paper. The samples were placed in marked envelopes and baked at 105°C for 30 minutes. Then the samples were dried to a constant weight of 65°C. The biomass of *Salix matsudana* seedings was determined by an analytical balance with an accuracy of 0.001 g (Li et al., 2022; Weng et al., 2022).

### 2.3 Determination of calcium concentration in leaves of *Salix matsudana* seedlings

The calcium content of plant leaves was determined using the method described by Miyazawa et al. (1984). A 0.15 g plant sample was placed in a decoction tube, and 6 ml of HNO_3_ and 2 ml of HClO_4_ were added. The drugs were left to react fully with the sample. The decoction tube was heated in the oven until the tissue inside the box turned white, and a large amount of white smoke appeared. The decoction tube was removed and cooled to room temperature, and the liquid was transferred to a 50 ml volumetric flask. 1.5 ml of 30% SrCl_2_ was added to the volumetric flask, and the final volume was fixed. The calcium content was determined using an atomic absorption spectrophotometer (AAS, Hitachi Z2000, Japan).

### 2.4 Determination of the photosynthetic pigments of *Salix matsudana* seedlings

Fresh leaves (0.1 g) were removed, shredded and extracted with 95% alcohol. A modified Arnon (1949) method, in which the absorption of light at wavelengths of 665 nm, 649 nm, and 479 nm of the cleaning solution was recorded, was used to determine the carotenoid, chlorophyll a, and chlorophyll b contents. The pigment contents were calculated according to the following equations (Li et al., 2022; Weng et al., 2022):


(Equation 1) Ca=13.95(A665)−6.88(A649)

(Equation 2) Cb=24.96(A649)− 7.32(A665)

(Equation 3) Cx=(1000(A479)−2.05Ca−114.8Cb)/245

(Equation 4) $$\text{Chlorophyll a}\left(\text{mg}\cdot \;{\text{kg}}^{-1}\right)=\left(\text{Ca}\times \text{Vt}\right)\left(\text{FW}\times 1000\right)\times \text{n}$$

(Equation 5) $$\text{Chlorophyll b}\left(\text{mg}\cdot {\text{kg}}^{-1}\right)=\left(\text{Cb}\times \text{Vt}\right)\left(\text{FW}\times 1000\right)\times \text{n}$$

(Equation 6) $$\text{Content of carotenoids}\left(\text{mg}\cdot {\text{kg}}^{-1}\right)=\left(\text{Cx}\times \text{Vt}\right)\left(\text{FW}\times 1000\right)\times \text{n}$$

where Ca, Cb and Cx are chlorophyll a, chlorophyll b and carotenoids, respectively. A665, A649, and A479 represent the absorbance values of the photosynthetic pigment extracts at 665, 649 and 479 nm, respectively. FW is the fresh weight of the sample, Vt is the total volume of extract, and n is the dilution factor.

### 2.5 Determination of photosynthetic parameters of *Salix matsudana* seedlings

The leaf net photosynthesis (Pn), transpiration (Tr) and stomatal conductance (Gs) values of the plants were measured with an LI-COR 6400 system (LI-COR Inc., Lincoln, NE, USA) between 10:00 and 12:00 on each sampling day. The effective light intensity was set to 1000 μmol·m^-2^·s^-1^, and the measurements were repeated 3 times. The most stable group of all measured data was selected for analysis (Li et al., 2022).

### 2.6 Determination of photosynthate of *Salix matsudana* seedlings

The soluble sugar and starch contents of *Salix matsudana* seedlings were determined using a UV-8000 spectrophotometer (Yuanxi, Beijing, China) based on spectrophotometry described in Doan et al. (2019) and Li et al. (2022). A total of 0.5 g of plant sample was placed in a centrifuge tube, and 10 ml of ethanol at 80% concentration was added. The mixture was placed in a hot water bath at 95°C for 10 minutes and then centrifuged at 5000 rpm for 10 minutes. The extraction process was repeated three times to ensure that all sugars were extracted, and all supernatants were combined and stored. To 0.2 ml of the soluble sugar extract, 5 ml of anthrone reagent was added, the mixture was watered in boiling water for 10 minutes and then cooled to room temperature, and the absorbance at 620 nm was measured to calculate the concentration of soluble sugars. The precipitate from the finished extraction was added to 3 ml of H_2_O, followed by a water bath in boiling water for 15 mins, and then after cooling, a total of 2 ml of 9.2 mol·L^-1^, 4.6 mol·L^-1^ HClO_4_ and H_2_O was added. The supernatant was extracted by centrifugation at 4000 rpm for 10 minutes. Water was added to the supernatant to achieve a constant volume of 50 ml, and absorbance was measured at 620 nm using the anthrone method. The starch concentration was then calculated from the standard curve for glucose.

### 2.7 Determination of the fluorescence parameters of *Salix matsudana* seedlings

Measurement of chlorophyll fluorescence of *Salix matsudana* seedlings was performed using a portable pulse-modulated chlorophyll fluorescence meter (OS-5P +, USA) based on the method described in Li et al. (2022).

### 2.8 Determination of the water use efficiency of *Salix matsudana* seedlings

The oven-dried leaves were ground through a 100-mesh sieve with a ball mill (Retsch200, Germany), and their δ^13^C values were measured by a stable isotope mass spectrometer (Isoprime100, UK) (Song et al., 2015; Li et al., 2022). The iWUE values were calculated from the following equation:


$$\text{iWUE}=\text{A}/\text{Gs}=\left(\text{Ca}-\text{Ci}\right)/1.6=\text{Ca}\left(1\;-\text{Ci}/\text{Ca}\right)/1.6=\text{Ca}\left(\text{b}-\delta {}^{13}\text{C}\right)/1.6\left(\text{b}-\text{a}\right)$$

where A is the net photosynthetic rate; Gs is the stomatal conductance; Ca and Ci are the CO_2_ pressure values in the atmosphere and leaf cells, respectively; and a and b are the partial effect of CO_2_ diffusion into the stomata and partial effect of stomatal photosynthetic carboxylase RUBP on carbon isotopes, respectively.

### 2.9 Determination of the antioxidant enzymes of *Salix matsudana* seedlings

For the determination of antioxidant enzymes, 0.4 g samples were taken from fresh leaves, stored in a frozen pipe, fixed with liquid nitrogen and stored at -80 °C. At the time of measurement, samples were taken according to the mark and put into a mortar. Then, 5 ml precooled phosphate buffer was added, the sample was ground, and homogenized pulp was centrifuged at four °C for 15 mins at 13000 rpm. The supernatant was placed into a centrifuge tube for reserve (3 repeats for each sample). The peroxidase (POD) level was determined by the guaiacol method. The catalase (CAT) level was determined by the ultraviolet absorption of hydrogen peroxide. Superoxide dismutase (SOD) levels were determined by methionine (Perveen et al., 2020; Ezzat et al., 2021).

### 2.10 Statistical analysis

Classification, mapping, statistical analysis, and significance analysis were performed using Excel and SPSS 22.0 software. The effects of different calcium and NaCl treatments on the growth and physiological characteristics of *Salix matsudana* seedlings were analyzed by Duncan’s new multiple extreme difference method. All experiments were replicated three times, and the results are expressed as the mean ± standard error (SE). The different letters in the chart indicate that the difference in each index between different treatments reached the significance level of 5%.

## 3 Results

### 3.1 Growth indices of *Salix matsudana* seedlings

**Figure 1:**
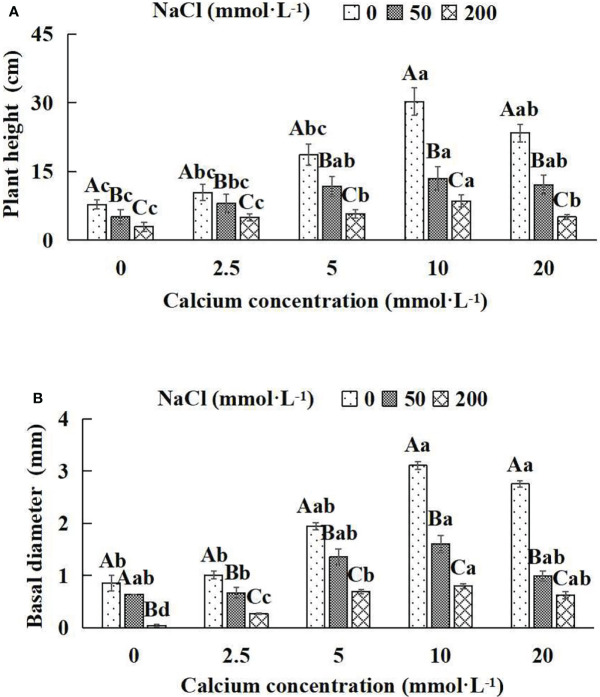
The growth indices of *Salix matsudana* seedlings under diverse treatments. Every column shows the mean ± SE, n = 3. Diverse lowercase letters indicate significant differences in the various calcium conditions for the same NaCl concentration. Diverse capital letters indicate significant differences in the various NaCl concentrations for the same calcium condition (*P<* 0.05). **(A)** Plant height; **(B)** Basal diameter.

In general, the indicators of growth and biomass of *Salix matsudana* seedlings showed decreasing trends with increasing NaCl concentrations (**Figures 1A, B**; **Table 1**). With the application of exogenous calcium, *Salix matsudana* seedlings were relieved from salt stress. The best alleviation was achieved when the exogenous calcium was 10 mmol·L^-1^, and the growth of *Salix matsudana* seedlings under different NaCl concentrations reached its maximum and was significantly different from other treatments (*P<* 0.05).

**Table 1:** Biomass of *Salix matsudana* seedlings under diverse treatments.

NaCl treatment(mmol·L^-1^)	Ca treatment(mmol·L^-1^)	Leaf biomass(g)	Stem biomass(g)	Lateral biomass(g)	Root biomass(g)	Total biomass(g)
0	0	6.88 ± 0.432Ad	5.58 ± 0.115Ac	6.67 ± 0.204Ac	4.86 ± 0.212Ac	23.99 ± 0.471Ae
	2.5	8.78 ± 0.259Acd	6.70 ± 0.078Abc	6.83 ± 0.191bBc	5.14 ± 0.087Bc	27.44 ± 0.228Ade
	5	10.22 ± 0.873Abc	6.89 ± 0.283Abc	7.13 ± 0.206Abc	6.02 ± 0.445Abc	30.26 ± 1.692Ad
	10	18.18 ± 0.522Aa	10.94 ± 1.082Aa	9.63 ± 0.503Aa	8.00 ± 0.629Aa	46.75 ± 0.650Aa
	20	11.91 ± 0.752Ab	7.74 ± 0.024Ab	8.25 ± 0.751Ab	7.26 ± 0.608Aab	35.15 ± 1.942Ab
50	0	6.80 ± 0.178Ab	5.30 ± 0.200Ac	5.82 ± 0.052Bb	4.91 ± 0.269Ac	22.83 ± 0.676Bbc
	2.5	7.62 ± 0.059Bb	6.25 ± 0.017Aab	7.15 ± 0.242Aa	5.61 ± 0.196Abc	26.63 ± 0.284Bb
	5	9.48 ± 0.425Ba	6.36 ± 0.463Bab	6.71 ± 0.43Bab	6.17 ± 0.666Aab	28.72 ± 1.945Bab
	10	10.16 ± 0.435Ba	7.01 ± 0.247Ba	6.74 ± 0.400Bab	7.07 ± 0.325Ba	30.98 ± 1.325Ba
	20	7.54 ± 0.165Bb	6.11 ± 0.166Cbc	7.31 ± 0.289Ba	6.75 ± 0.251Bab	27.71 ± 0.398Bab
200	0	6.42 ± 0.040Bc	5.14 ± 0.027Ab	5.10 ± 0.022Cc	3.90 ± 0.037Bb	20.56 ± 0.052Cd
	2.5	6.78 ± 0.042Cc	5.16 ± 0.077Bb	5.71 ± 0.136Cbc	5.69 ± 0.077Aa	23.34 ± 0.281Cc
	5	7.64 ± 0.17C4b	6.58 ± 0.079Ba	6.34 ± 0.304Bab	6.00 ± 0.075Aa	26.55 ± 0.370Cb
	10	8.50 ± 0.265Ca	7.31 ± 0.275Ba	6.70 ± 0.206Ba	7.18 ± 0.966Ba	29.69 ± 0.937Ba
	20	6.83 ± 0.082Bc	6.93 ± 0.483Ba	6.56 ± 0.324Ca	6.21 ± 0.136Ba	26.53 ± 0.936Bb

Every column and table show the mean ± SE, n = 3. Diverse lowercase letters indicate significant differences between treatments of calcium addition under the same NaCl concentration. Diverse capital letters indicate significant differences in the various NaCl concentrations for the same calcium condition (P< 0.05).

When salt stress did not occur, the height, basal diameter and total biomass of *Salix matsudana* seedlings reached maximum values of 30.23 cm, 3.10 mm and 46.75 g, respectively, at 10 mmol·L^-1^ exogenous calcium. The increase was 290.00%, 263.04% and 94.87% compared to that without calcium application. At a NaCl concentration of 50 mmol·L^-1^, the height, basal diameter and total biomass of *Salix matsudana* seedlings were inhibited, decreasing by 34.84%, 25.25% and 4.84%, respectively, compared to those without calcium. At an exogenous calcium concentration of 10 mmol·L^-1^, the salt stress of *Salix matsudana* seedlings was reduced to 13.48 cm, 1.60 mm and 30.98 g, respectively. The increase was 166.93%, 149.69% and 35.70% compared to that without calcium application. At a NaCl concentration of 200 mmol·L^-1^, the height, basal diameter and total biomass of *Salix matsudana* seedlings were more severely suppressed, decreasing by 62.58%, 95.35% and 16.68%, respectively, compared to those without NaCl stress. At a concentration of 10 mmol·L^-1^ exogenous calcium, *Salix matsudana* seedlings showed the greatest alleviation from salt stress at 8.55 cm, 0.80 mm and 29.69 g, respectively. The increases were 66.08%, 95.00% and 30.61% compared to those without calcium application (**Figures 1A, B**; **Table 1**).

With the increase in exogenous calcium content, the alleviation effect on the growth of *Salix matsudana* seedlings under salt stress showed an overall trend of increasing and then decreasing. The best alleviation effect was achieved when the exogenous calcium content was 10 mmol·L^-1^, and the growth effect of *Salix matsudana* reached the maximum.

### 3.2 Calcium concentration in leaves of *Salix matsudana* seedlings

The calcium concentration of *Salix matsudana* seedlings decreased significantly with increasing NaCl concentrations under five different exogenous calcium concentrations. There were remarkable differences in calcium content among different treatments (*P*< 0.05). With the application of exogenous calcium, *Salix matsudana* seedlings were relieved from salt stress. The best alleviation was achieved when the exogenous calcium was 10 mmol·L^-1^, and the leaves’ calcium content of *Salix matsudana* seedlings under different NaCl concentrations reached its maximum and was significantly different from other treatments (*P*< 0.05).

**Figure 2:**
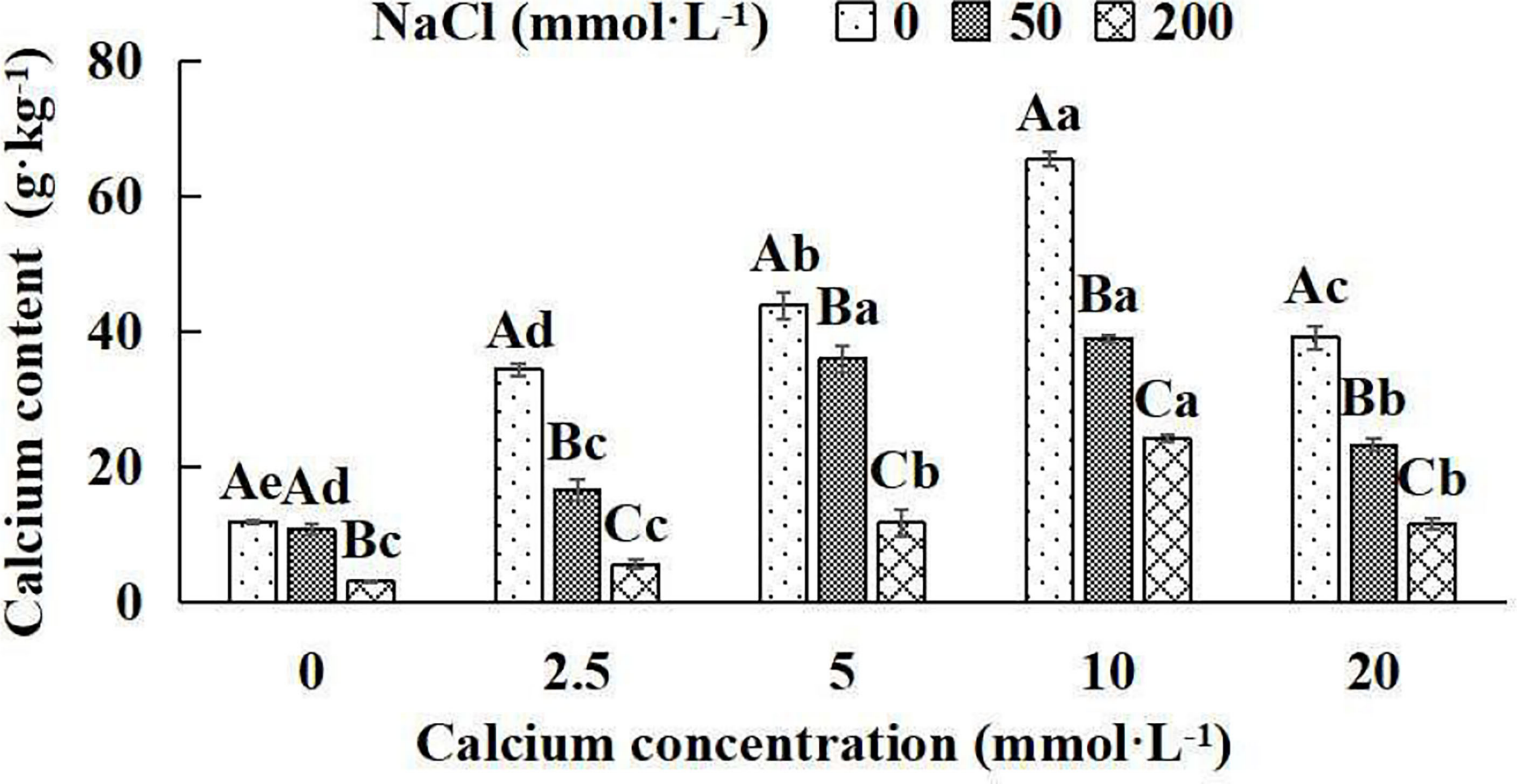
Calcium concentration in leaves of *Salix matsudana* seedlings under diverse treatment. Note: Every column shows the mean ± SE, n = 3. Diverse lowercase letters indicate significant differences in the various calcium conditions for the same NaCl concentration. Diverse capital letters indicate significant differences in the various NaCl concentrations for the same calcium condition (*P<* 0.05).

When salt stress did not occur, the leaves’ calcium content of *Salix matsudana* seedlings reached a maximum value of 65.31 g·kg^-1^, at 10 mmol·L^-1^ exogenous calcium. The increase was 81.96% compared to that without calcium application. At a NaCl concentration of 50 mmol·L^-1^, the leaves’ calcium content of *Salix matsudana* seedlings was inhibited, decreasing by 10.02%, compared to those without calcium. At an exogenous calcium concentration of 10 mmol·L^-1^, the leaves’ calcium content of *Salix matsudana* seedlings was increased to 38.88 g·kg^-1^. The increase was 266.79% compared to that without calcium application. At a NaCl concentration of 200 mmol·L^-1^, the leaves’ calcium content of *Salix matsudana* seedlings was more severely suppressed, decreasing by 75.13%, compared to those without NaCl stress. At a concentration of 10 mmol·L^-1^ exogenous calcium, *Salix matsudana* seedlings showed the greatest alleviation from salt stress at 24.02 g·kg^-1^. The increase was 719.80% compared to that without calcium application (**Figure 2**).

With the increase in exogenous calcium content, the leaves’ calcium content of *Salix matsudana* seedlings under salt stress showed an overall trend of increasing and then decreasing. The leaves’ calcium content of *Salix matsudana* achieved the maximum when the exogenous calcium content was 10 mmol·L^-1^.

### 3.3 The Photosynthetic pigments of *Salix matsudana* seedlings

**Figure 3:**
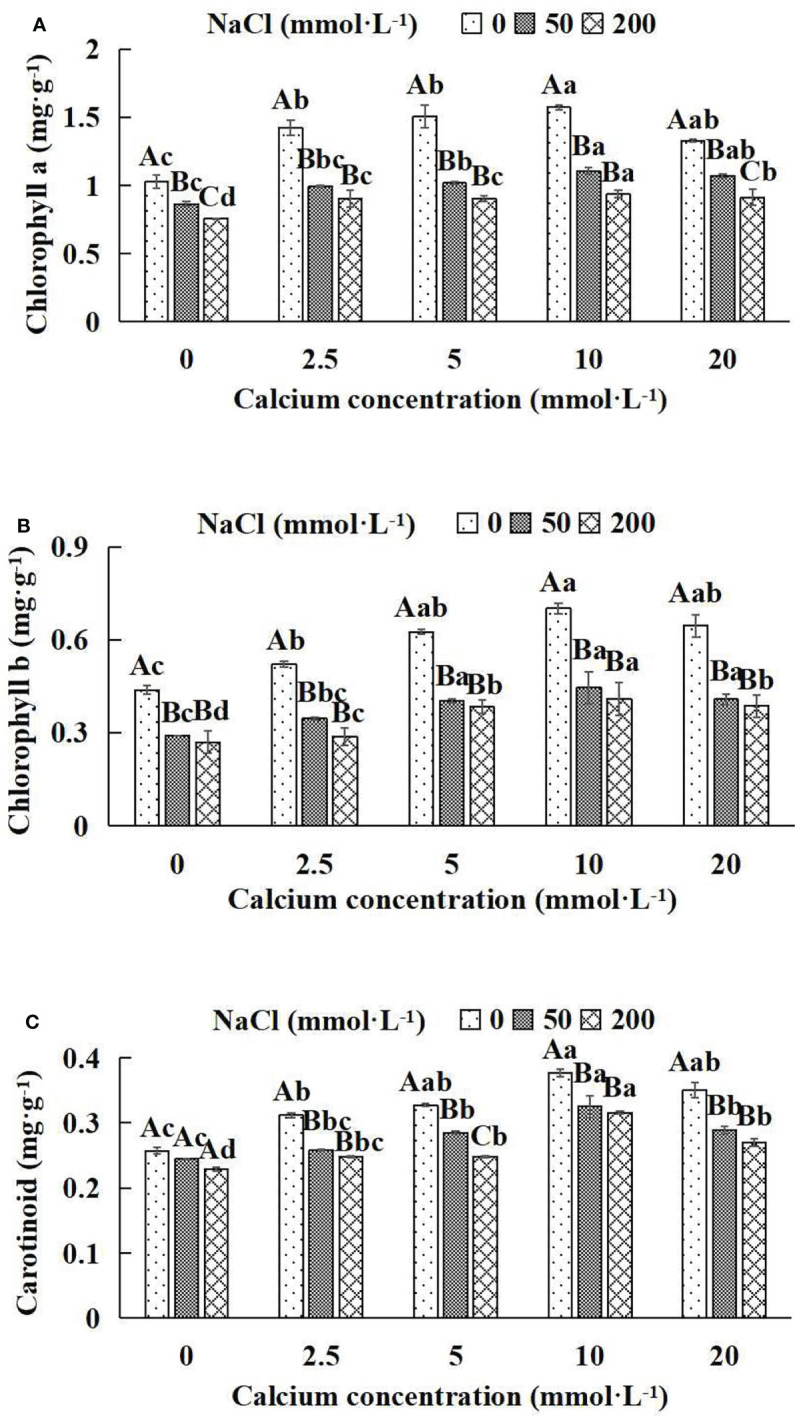
Photosynthetic pigments of *Salix matsudana* seedlings under diverse treatments. Every column shows the mean ± SE, n = 3. Diverse lowercase letters indicate significant differences in the various calcium conditions for the same NaCl concentration. Diverse capital letters indicate significant differences in the various NaCl concentrations for the same calcium condition (*P<* 0.05). **(A)** Chlorophyll a; **(B)** Chlorophyll b; **(C)** Carotinoid.

In general, at the same NaCl concentration, photosynthetic pigment indices (chlorophyll a, chlorophyll b, carotenoid) increased and then decreased with the increase of calcium concentrations (**Figures 3A–C**). With the application of exogenous calcium, *Salix matsudana* seedlings were relieved from salt stress. The best alleviation was achieved when the exogenous calcium was 10 mmol·L^-1^, and the photosynthetic pigment indices (Chlorophyll a, chlorophyll b and carotenoid) of *Salix matsudana* seedlings under different NaCl concentrations reached their maximum and were significantly different from other treatments (*P*< 0.05).

When salt stress did not occur, the chlorophyll a, chlorophyll b and carotenoid of *Salix matsudana* seedlings reached maximum values of 1.57, 0.70 and 0.38 mg·g^-1^, respectively, at 10 mmol·L^-1^ exogenous calcium. The increase was 53.92%, 59.09% and 46.15% compared to that without calcium application. At a NaCl concentration of 50 mmol·L^-1^, the chlorophyll a, chlorophyll b and carotenoid of *Salix matsudana* seedlings were inhibited, decreasing by 16.50%, 34.09% and 7.69%, respectively, compared to those without calcium. At an exogenous calcium concentration of 10 mmol·L^-1^, the chlorophyll a, chlorophyll b and carotenoid of *Salix matsudana* seedlings were maximum increased to 1.11, 0.44 and 0.33 mg·g^-1^, respectively. The increase was 29.07%, 51.72% and 37.50% compared to that without calcium application. At a NaCl concentration of 200 mmol·L^-1^, the chlorophyll a, chlorophyll b and carotenoid of *Salix matsudana* seedlings were more severely suppressed, decreasing by 26.21%, 38.64% and 11.54%, respectively, compared to those without NaCl stress. At a concentration of 10 mmol·L^-1^ exogenous calcium, *Salix matsudana* seedlings showed alleviation from salt stress at 0.94, 0.41 and 0.32 mg·g^-1^, respectively. The increases were 23.68%, 51.85% and 39.13% compared to those without calcium application (**Figures 3A–C**).

With the increase in exogenous calcium content, the alleviation effect on the photosynthetic pigment of *Salix matsudana* seedlings under salt stress showed an overall trend of increasing and then decreasing. The best alleviation effect was achieved when the exogenous calcium content was 10 mmol·L^-1^, and the chlorophyll a, chlorophyll b and carotenoid effect of *Salix matsudana* reached the maximum.

### 3.4 Photosynthetic parameters of *Salix matsudana* seedlings

**Figure 4:**
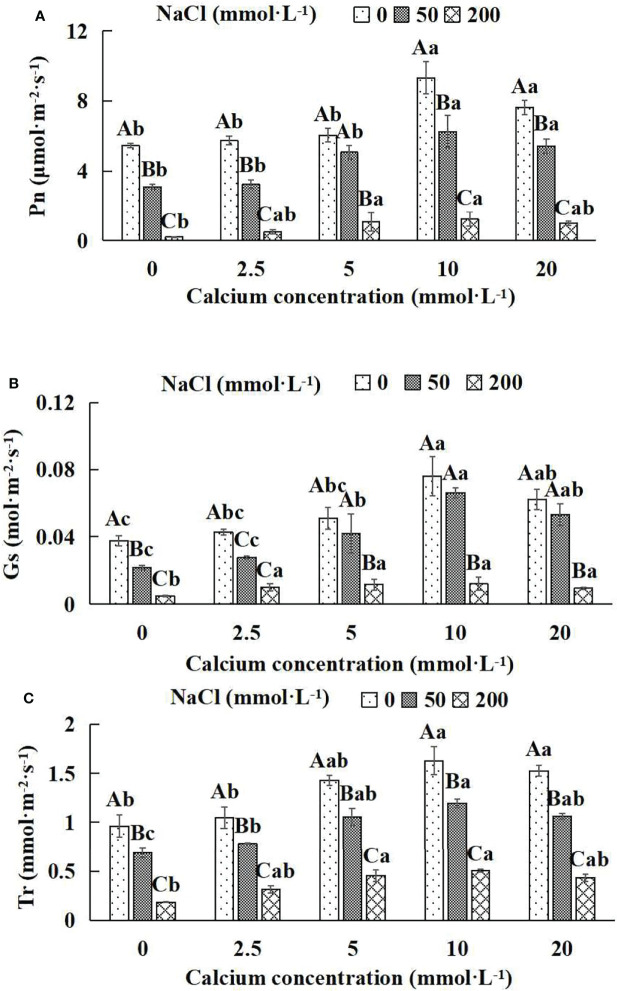
Photosynthetic parameters of *Salix matsudana* seedlings under diverse treatments. Note: Every column shows the mean ± SE, n = 3. Diverse lowercase letters indicate significant differences in the various calcium conditions for the same NaCl concentration. Diverse capital letters indicate significant differences in the various NaCl concentrations for the same calcium condition (*P<* 0.05). **(A)** Pn (Photosynthetic rate); **(B)** Gs (Conductance to H_2_O); **(C)** Tr (Transpiration rate).

In general, the Photosynthetic parameters (Pn, Tr and Gs) increased and then decreased with the increase of the exogenous calcium concentrations at the same NaCl concentration (**Figures 4A–C**). With the application of exogenous calcium, *Salix matsudana* seedlings were relieved from salt stress. The best alleviation was achieved when the exogenous calcium was 10 mmol·L^-1^, and the Photosynthetic parameters of *Salix matsudana* seedlings under different NaCl concentrations reached their maximum and were significantly different from other treatments (*P*< 0.05).

When salt stress did not occur, the Pn, Gs and Tr of *Salix matsudana* seedlings reached maximum values of 9.33 μmol·m^-2^·s^-1^, 0.08 mol·m^-2^·s^-1^ and 1.63 mmol·m^-2^·s^-1^, respectively, at 10 mmol·L^-1^ exogenous calcium. The increase was 71.51%, 100.00% and 69.79% compared to that without calcium application. At a NaCl concentration of 50 mmol·L^-1^, the Pn, Gs and Tr of *Salix matsudana* seedlings were inhibited, decreasing by 42.83%, 50.00% and 28.13%, respectively, compared to those without calcium. At an exogenous calcium concentration of 10 mmol·L^-1^, the Pn, Gs and Tr of *Salix matsudana* seedlings were increased to 6.24 μmol·m^-2^·s^-1^, 0.07 mol·m^-2^·s^-1^ and 1.19 mmol·m^-2^·s^-1^, respectively. The increase was 100.64%, 250.00% and 72.46% compared to that without calcium application. At a NaCl concentration of 200 mmol·L^-1^, the Pn, Gs and Tr of *Salix matsudana* seedlings were more severely suppressed, decreasing by 95.96%, 87.23% and 80.73%, respectively, compared to those without NaCl stress. At a concentration of 10 mmol·L^-1^ exogenous calcium, *Salix matsudana* seedlings showed the greatest alleviation from salt stress at 1.25 μmol·m^-2^·s^-1^, 0.01 mol·m^-2^·s^-1^ and 0.50 mmol·m^-2^·s^-1^. The increases were 468.18%, 150.00% and 172.43%, respectively, compared to those without calcium application (**Figures 4A–C**).

With the increase in exogenous calcium content, the alleviation effect on the Photosynthetic parameters of *Salix matsudana* seedlings under salt stress showed an overall trend of increasing and then decreasing. The best alleviation effect was achieved when the exogenous calcium content was 10 mmol·L^-1^, and the Pn, Gs and Tr of *Salix matsudana* reached the maximum.

### 3.5 Photosynthate of *Salix matsudana* seedlings

**Figure 5:**
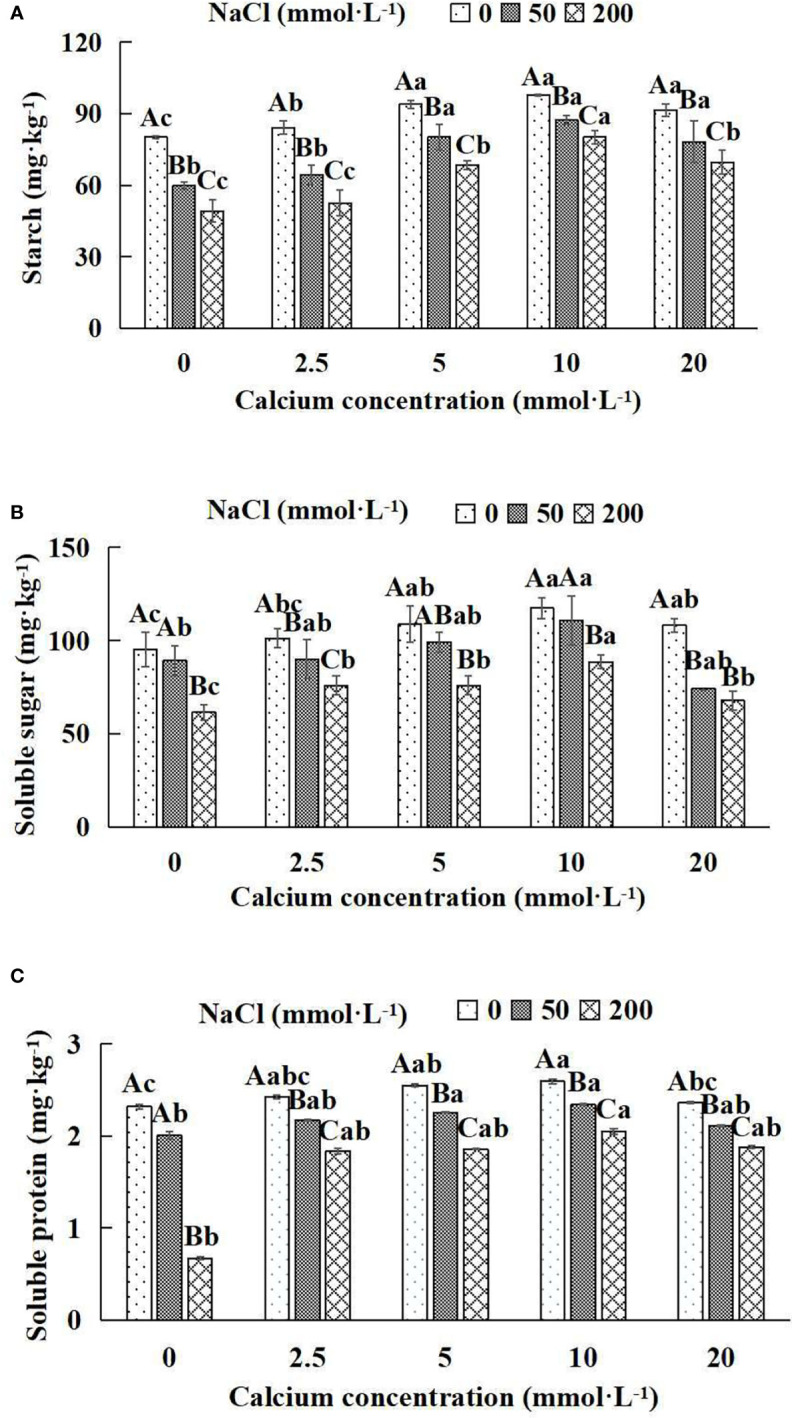
Photosynthates of *Salix matsudana* seedlings under diverse treatments. Note: Every column shows the mean ± SE, n = 3. Diverse lowercase letters indicate significant differences in the various calcium conditions for the same NaCl concentration. Diverse capital letters indicate significant differences in the various NaCl concentrations for the same calcium condition (*P<* 0.05). **(A)** Starch; **(B)** Soluble sugar; **(C)** Soluble protein.

In general, the indexes of the photosynthates (starch, soluble sugar and soluble protein) increased first and then decreased with the increase of the exogenous calcium concentrations at the same NaCl concentration (**Figures 5A–C**). With the application of exogenous calcium, *Salix matsudana* seedlings were relieved from salt stress. The best alleviation was achieved when the exogenous calcium was 10 mmol·L^-1^, and the photosynthates of *Salix matsudana* seedlings under different NaCl concentrations reached their maximum and were significantly different from other treatments (*P*< 0.05).

When salt stress did not occur, the starch, soluble sugar and soluble protein of *Salix matsudana* seedlings reached maximum values of 97.94, 117.42 and 2.59 mg·kg^-1^, respectively, at 10 mmol·L^-1^ exogenous calcium. The increase was 22.18%, 23.44% and 11.64% compared to that without calcium application. At a NaCl concentration of 50 mmol·L^-1^, the starch, soluble sugar and soluble protein of *Salix matsudana* seedlings were inhibited, decreasing by 25.46%, 6.32% and 13.79%, respectively, compared to those without calcium. At an exogenous calcium concentration of 10 mmol·L^-1^, the starch, soluble sugar and soluble protein of *Salix matsudana* seedlings were increased to 87.40, 110.69 and 2.34 mg·kg^-1^, respectively. The increase was 46.28%, 24.20% and 17.00%, compared to that without calcium application. At a NaCl concentration of 200 mmol·L^-1^, the starch, soluble sugar and soluble protein of *Salix matsudana* seedlings were more severely suppressed, decreasing by 38.60%, 35.42% and 71.12%, respectively, compared to those without NaCl stress. At a concentration of 10 mmol·L^-1^ exogenous calcium, *Salix matsudana* seedlings showed the greatest alleviation from salt stress at 80.16, 88.60 and 2.05 mg·kg^-1^, respectively. The increases were 62.86%, 44.23% and 205.97% compared to those without calcium application (**Figures 5A–C**).

With the increase in exogenous calcium content, the alleviation effect on the photosynthates of *Salix matsudana* seedlings under salt stress showed an overall trend of increasing and then decreasing. The best alleviation effect was achieved when the exogenous calcium content was 10 mmol·L^-1^, and the starch, soluble sugar and soluble protein of *Salix matsudana* reached the maximum.

### 3.6 The fluorescence parameters of *Salix matsudana* seedlings

**Figure 6:**
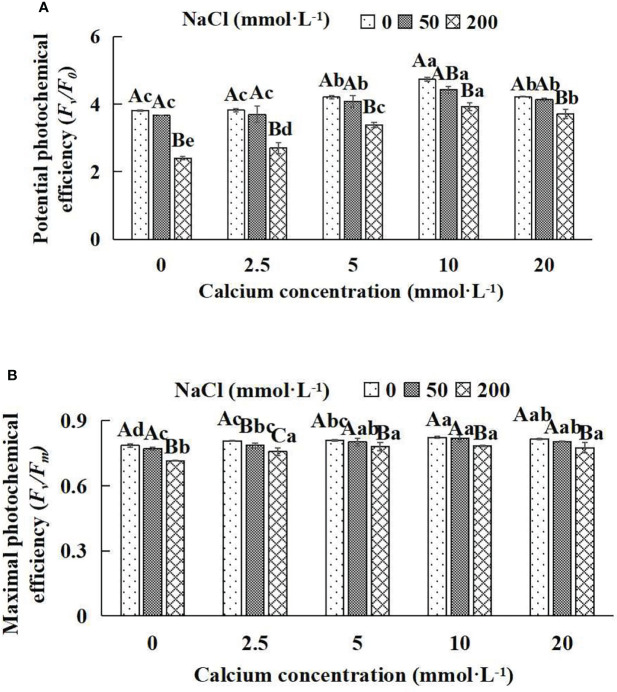
Fluorescence parameters of *Salix matsudana* seedlings under diverse treatments. Note: *F*_v_ */F*_0_ indicates the potential activity of PSII, and *F*_v_ */F*_m_ represents the maximum photochemical efficiency of PSII. Note: Diverse lowercase letters indicate significant differences in the various calcium conditions for the same NaCl concentration. Diverse capital letters indicate significant differences in the various NaCl concentrations for the same calcium condition (*P<* 0.05). **(A)** *F*_v_ */F*_0_ (Potential photochemical efficiency). **(B)** *F*_v_ */F*_m_ (Maximal photochemical efficiency).

In general, the fluorescence parameters of chlorophyll increased and then decreased with the increase of the exogenous calcium concentrations at the same NaCl concentration (**Figures 6A, B**). With the application of exogenous calcium, *Salix matsudana* seedlings were relieved from salt stress. The best alleviation was achieved when the exogenous calcium was 10 mmol·L^-1^, and the fluorescence parameters of *Salix matsudana* seedlings under different NaCl concentrations reached their maximum and were significantly different from other treatments (*P*< 0.05).

When salt stress did not occur, the *F*_v_ */F*_0_ and *F*_v_ */F*_m_ of *Salix matsudana* seedlings reached maximum values of 0.82 and 4.73, respectively, at 10 mmol·L^-1^ exogenous calcium. The increase was 4.71% and 24.15% compared to that without calcium application. At a NaCl concentration of 50 mmol·L^-1^, the *F*_v_ */F*_0_ and *F*_v_ */F*_m_ of *Salix matsudana* seedlings were inhibited, decreasing by 1.28% and 5.4%, respectively, compared to those without calcium. At an exogenous calcium concentration of 10 mmol·L^-1^, the fluorescence parameters of *Salix matsudana* seedlings were increased to 0.82 and 4.43. The increase was 5.96% and 20.71% compared to that without calcium application. At a NaCl concentration of 200 mmol·L^-1^, the *F*_v_ */F*_0_ and *F*_v_ */F*_m_ of *Salix matsudana* seedlings were more severely suppressed, decreasing by 8.86% and 37.01%, respectively, compared to those without NaCl stress. At a concentration of 10 mmol·L^-1^ exogenous calcium, *Salix matsudana* seedlings showed the greatest alleviation from salt stress at 0.78 and 3.93, respectively. The increases were 9.65% and 63.63%, respectively, compared to those without calcium application (**Figures 6A, B**).

With the increase in exogenous calcium content, the alleviation effect on the fluorescence parameters of *Salix matsudana* seedlings under salt stress showed an overall trend of increasing and then decreasing. The best alleviation effect was achieved when the exogenous calcium content was 10 mmol·L^-1^, and the *F*_v_ */F*_0_ and *F*_v_ */F*_m_ of *Salix matsudana* reached the maximum.

### 3.7 Water use efficiency of *Salix matsudana* seedlings

**Figure 7:**
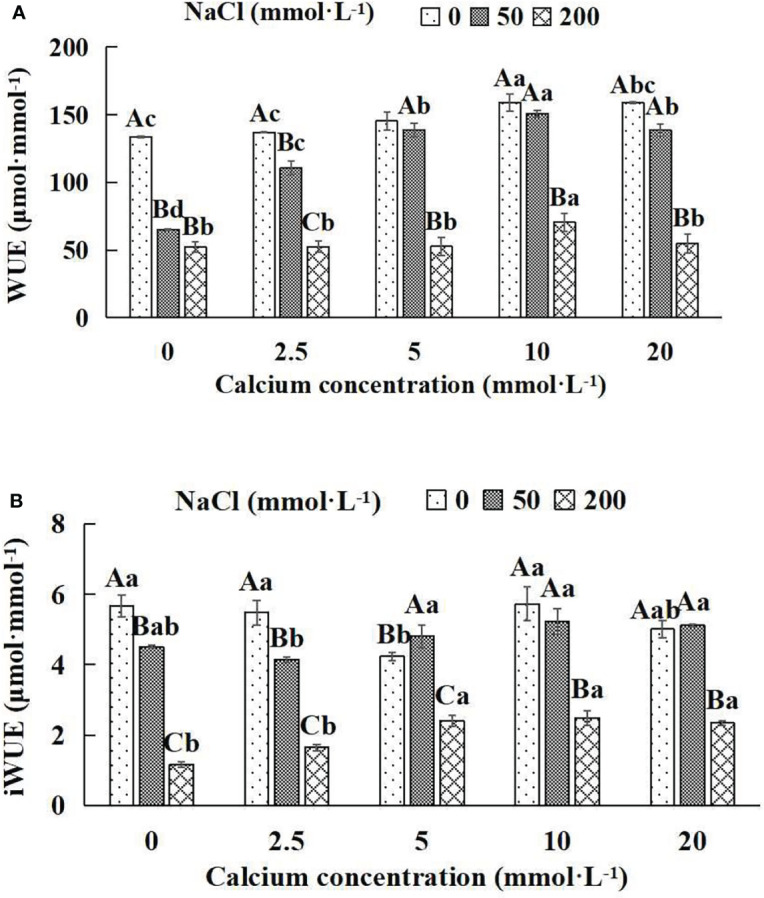
Water use efficiency of *Salix matsudana* seedlings under diverse treatments. Note: Every column shows the mean ± SE, n = 3. Diverse lowercase letters indicate significant differences in the various calcium conditions for the same NaCl concentration. Diverse capital letters indicate significant differences in the various NaCl concentrations for the same calcium condition (*P<* 0.05). **(A)** WUE (Instantaneous water use efficiency). **(B)** iWUE (Water use efficiency).

In general, the value of water use efficiency (WUE and iWUE) increased first and then decreased with the increase of exogenous calcium concentrations (**Figures 7A, B**). The maximum water use efficiency value occurred at 10 mmol·L^-1^ calcium concentration. The water use efficiency values of *Salix matsudana* seedlings under different NaCl concentrations were remarkably affected by adding diverse exogenous calcium concentrations. With the increase of exogenous calcium and NaCl concentrations, there was a significant difference between WUE and other treatments when the exogenous calcium concentration was 10 mmol·L^-1^ (*P*< 0.05).

When salt stress did not occur, the WUE and iWUE of *Salix matsudana* seedlings reached maximum values of 158.84 and 5.72 μmol·mmol^-1^ at 10 mmol·L^-1^ exogenous calcium. The increase was 18.97% and 0.88% compared to that without calcium application. At a NaCl concentration of 50 mmol·L^-1^, the WUE and iWUE of *Salix matsudana* seedlings were inhibited, decreasing by 51.30% and 20.46%, respectively, compared to those without calcium. At an exogenous calcium concentration of 10 mmol·L^-1^, the WUE and iWUE of *Salix matsudana* seedlings were increased to 150.60 and 5.23 μmol·mmol^-1^. The increase was 131.62% and 15.96% compared to that without calcium application. At a NaCl concentration of 200 mmol·L^-1^, the WUE and iWUE of *Salix matsudana* seedlings were more suppressed, decreasing by 60.74% and 79.54%, respectively, compared to those without NaCl stress. At a concentration of 10 mmol·L^-1^ exogenous calcium, *Salix matsudana* seedlings showed alleviation from salt stress at 70.56 and 2.49 μmol·mmol^-1^. The increases were 34.63% and 114.66% compared to those without calcium application (**Figures 7A, B**).

With the increase in exogenous calcium content, the alleviation effect on water use efficiency of *Salix matsudana* seedlings under salt stress showed an overall trend of increasing and then decreasing. The best alleviation effect was achieved when the exogenous calcium content was 10 mmol·L^-1^, and the WUE and iWUE of *Salix matsudana* reached the maximum.

### 3.8 The antioxidant enzymes of *Salix matsudana* seedlings

**Figure 8:**
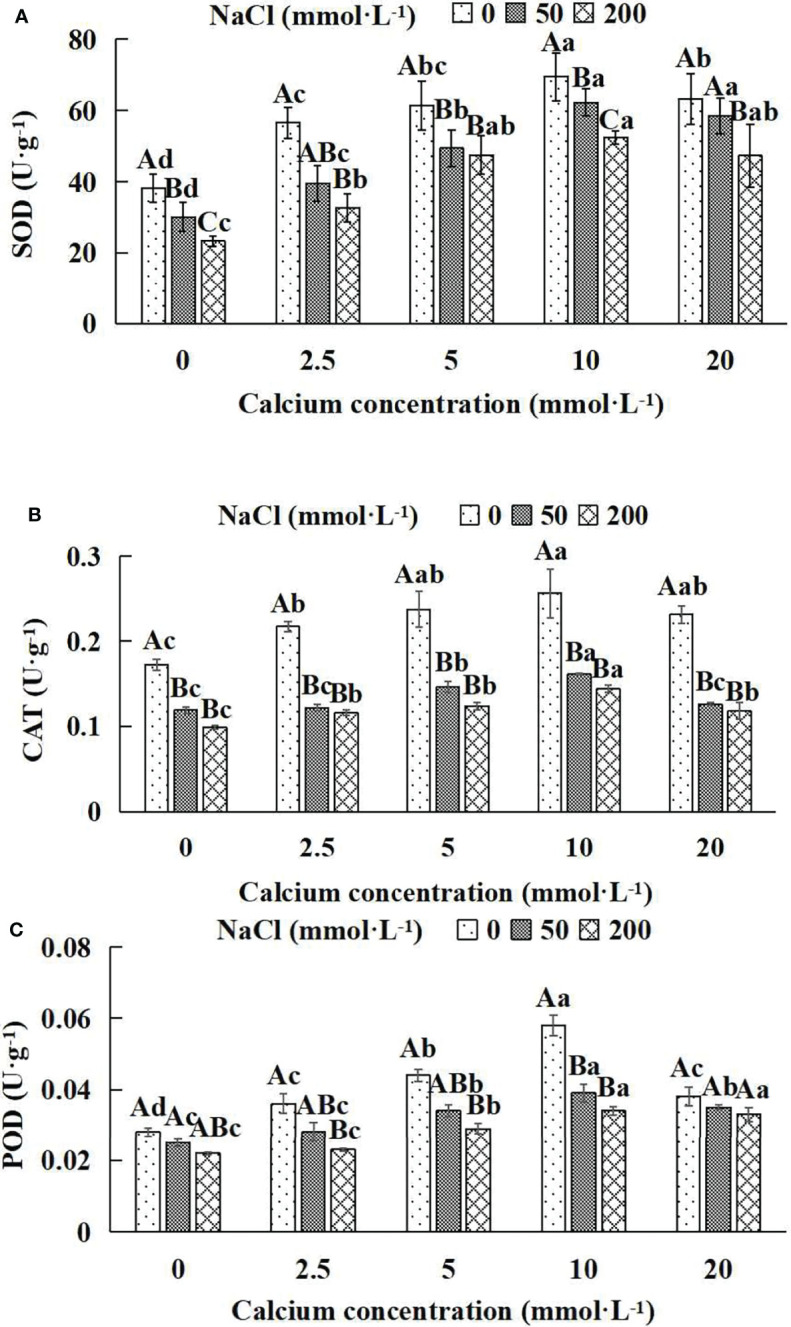
Antioxidant enzymes of *Salix matsudana* seedlings under diverse treatments. Note: CAT represents catalase, SOD represents superoxide dismutase and POD represents peroxidase. Note: Every column shows the mean ± SE, n = 3. Diverse lowercase letters indicate significant differences in the various calcium conditions for the same NaCl concentration. Diverse capital letters indicate significant differences in the various NaCl concentrations for the same calcium condition (*P<* 0.05). **(A)** SOD (Superoxide dismutase); **(B)** CAT (Catalase); **(C)** POD (Peroxidase).

In general, the activities of the antioxidant enzyme increased first and then decreased with the increase of exogenous calcium concentrations (**Figures 8A–C**). The maximum values of SOD, CAT and POD occurred under the 10 mmol·L^-1^ calcium concentration. The antioxidant enzyme activities of *Salix matsudana* seedlings under different NaCl concentrations were remarkably affected by adding diverse exogenous calcium concentrations. With the application of exogenous calcium, *Salix matsudana* seedlings were relieved from salt stress. The best alleviation was achieved when the exogenous calcium was 10 mmol·L^-1^, and the activity of the antioxidant enzyme of *Salix matsudana* seedlings under different NaCl concentrations reached its maximum and was significantly different from other treatments (*P*< 0.05).

When salt stress did not occur, the SOD, CAT and POD of *Salix matsudana* seedlings reached maximum values of 69.5, 0.256 and 0.058 U·g^-1^, respectively, at 10 mmol·L^-1^ exogenous calcium. The increase was 81.46%, 48.84% and 107.14% compared to that without calcium application. At a NaCl concentration of 50 mmol·L^-1^, the SOD, CAT and POD of *Salix matsudana* seedlings were inhibited, decreasing by 21.41%, 30.81% and 10.71%, respectively, compared to those without calcium. At an exogenous calcium concentration of 10 mmol·L^-1^, the SOD, CAT and POD of *Salix matsudana* seedlings were increased to 62.3, 0.161 and 0.039 U·g^-1^, respectively. The increase was 106.98%, 35.29% and 56.00% compared to that without calcium application. At a NaCl concentration of 200 mmol·L^-1^, the SOD, CAT and POD of *Salix matsudana* seedlings were more severely suppressed, decreasing by 39.16%, 42.44% and 27.27%, respectively, compared to those without NaCl stress. At a concentration of 10 mmol·L^-1^ exogenous calcium, *Salix matsudana* seedlings showed the greatest alleviation from salt stress at 52.4, 0.144 and 0.034 U·g^-1^, respectively. The increases were 124.89%, 45.45% and 54.55% compared to those without calcium application (**Figure 8A–C**).

With the increase in exogenous calcium content, the alleviation effect on the SOD, CAT and POD of *Salix matsudana* seedlings under salt stress showed an overall trend of increasing and then decreasing. The best alleviation effect was achieved when the exogenous calcium content was 10 mmol·L^-1^, and the SOD, CAT and POD of *Salix matsudana* reached the maximum.

## 4 Discussion

### 4.1 Exogenous calcium regulates the growth of *Salix matsudana* seedlings under NaCl stress

As one of the most severe sources of pollution in the world, salt stress affects plant metabolism and slows plant growth. The salt tolerance of different plant species has been demonstrated by studying the growth response of plants under other salt concentration treatments (Goyal et al., 2018; Safdar et al., 2019). The accumulation of small amounts of salt in cells disrupts normal physiological and biochemical processes in plants, which in turn inhibits plant growth (Parihar et al., 2014; Yang and Guo, 2017; Goyal et al., 2018). Munns (2005) found that plant roots are the first to perceive salt stress and rapidly impair plant growth by reducing water supply-induced osmotic pressure, leading to a nutrient imbalance in the cell membrane. Salt stress causes stomata to close, CO_2_ cannot be fixed, and the normal electron flow for carbon reduction in the Calvin cycle is disturbed (Acosta-Motos et al., 2017). Under salt stress conditions, the growth and biomass of alfalfa crops and rice are significantly reduced (Mbarki et al., 2018; Roy et al., 2019). Elkelish et al. (2019) also confirmed that especially in calcium-deficient plants under salt stress, plant height and biomass growth are reduced. In this experiment, the height, basal diameter and biomass of *Salix matsudana* seedlings decreased to different degrees with increasing NaCl concentrations. Ca^2+^-induced ion channels distinguish Na^+^ (Schroeder and Hagiwara, 1989), contribute to the composition of cell walls and cell membranes and are balanced and stabilized by contact with different proteins and lipids on the cell membrane surface (Davis et al., 2003; Roy et al., 2019). Therefore, Ca^2+^ can mitigate plant growth under salt stress conditions (Kurth et al., 1986). Tuna et al. (2007) found that the application of exogenous calcium improved the inhibition of plant growth by salt stress. The addition of calcium has been shown effective in promoting the growth and yield of tomato, soybean and cucumber under salt stress (Navarro et al., 2000; Elkelish et al., 2019; Wang et al., 2021). The leaf area, biomass and roots of salt-tolerant and salt-sensitive rice have been shown to increase significantly with increasing exogenous calcium (Cha-um et al., 2012). Lei et al. (2013) found that Ca supplementation significantly alleviated the inhibitory effect of NaCl stress on pumpkin rootstock graft growth. Earlier studies have shown that the application of exogenous Ca alleviates the development of cowpeas, *Rumex*, Jerusalem artichoke, loquat and anger rootstocks, linseed and bean under salt stress (Murillo-Amador et al., 2006; Chen et al., 2006; Xue et al., 2008; García-Legaz et al., 2008; Khan et al., 2009; Cabot et al., 2009). Exogenous calcium also reduces leaf wilt of salt-stressed date sour jujube by nearly half (Jin et al., 2016). Our study showed that the growth and biomass of *Salix matsudana* seedlings decreased with increasing NaCl concentration. Exogenous calcium could improve the growth and biomass of *Salix matsudana* seedlings under salt stress, showing a trend of increasing first and then decreasing. When the concentration of exogenous calcium was 10 mmol·L^-1^, the height, basal diameter and biomass of *Salix matsudana* seedlings reached their highest values. In conclusion, the optimal concentration of exogenous calcium for alleviating salt stress and promoting the growth of *Salix matsudana* seedlings was 10 mmol·L^-1^.

### 4.2 Exogenous calcium regulates the photosynthetic characteristics of *Salix matsudana* seedlings under NaCl stress

Salt stress causes a reduction in plant stomatal conductance and chlorophyll content and reduced transpiration or reduced efficiency of photosystem II, resulting in difficulties in transporting ions from the soil to the root surface, which in turn affects plant photosynthesis (Netondo et al., 2004; Guo et al., 2021). Previous studies have shown that salt stress affects plant photosynthesis, with chlorophyll and carotenoid contents decreasing as the salt content in young leaves increases (Parida et al., 2002; Stepien and Johnson, 2008; Duarte et al., 2013). Chlorophyll a, chlorophyll b and total chlorophyll were significantly reduced in pea leaves and rice seedlings under salt stress (Ozturk et al., 2012; Zhen-hua et al., 2012; Roy et al., 2019). Under salt stress conditions, plants reduce the flow of toxic ions in the transpiration stream by closing stomata (Kerstiens et al., 2002; Veselov et al., 2008; Vysotskaya et al., 2010), reducing osmoregulatory solutes and increasing ROS in chloroplasts, leading to a decrease in plant photosynthesis (Xu et al., 2017). Earlier studies found that salt stress reduced stomatal conductance in Euonymus plants, Viburnum tinus and Metrosideros excelsa (Bañón et al., 2012; Gómez et al., 2013). Tang et al. (2014) found that plant growth was closely related to photosynthetic pigments and the photosynthetic rate and that increased chlorophyll degradation caused by salt stress resulted in a decrease in plant photosynthetic efficiency (Elkelish et al., 2019). The reduction in chlorophyll content of wheat seedlings and *Artemisia annua* L. exposed to salt stress is due to the oxidation of chlorophyll and other chloroplast pigments and the destabilization of pigment-protein complexes (Aftab et al., 2011; Tian et al., 2015). Rahman et al. (2016) suggested that under salt stress, Ca^2+^ addition can increase chlorophyll by producing limited ROS. When soybean seedlings are exposed to salt stress, chlorophyllase activity rises, and the synthesis of chlorophyll intermediates decreases (Elkelish et al., 2019). Our findings are in agreement with previous work showing that the photosynthetic pigments, photosynthetic products and chlorophyll fluorescence of *Salix matsudana* seedlings gradually decrease with increasing NaCl concentration. Ca^2+^ application affects most physiological processes in salt-stressed plants and regulates photosynthetic pigment content (Roy et al., 2019; Lu et al., 2021). Xu et al. (2013) found that CaCl_2_ application increased chlorophyll synthesis and chlorophyll fluorescence as well as increased photosynthetic rates in *Zoysia japonica*. Under salt stress, the addition of Ca^2+^ increased chlorophyll content, and the presence of optimal Ca concentrations significantly improved photosynthetic pigment accumulation, resulting in enhanced photosynthetic efficiency and stomatal conductance (Elkelish et al., 2019). Studies have demonstrated that the application of Ca^2+^ increases the total chlorophyll content of salt-affected rice and tartary buckwheat cultivars (Roy et al., 2019; Lu et al., 2021). Our study showed that the photosynthetic characteristics of *Salix matsudana* seedlings decreased with increasing NaCl concentration. Exogenous calcium could improve the photosynthetic characteristics of *Salix matsudana* seedlings under salt stress, showing a trend of increasing first and then reducing. When the concentration of exogenous calcium was 10 mmol·L^-1^, the chlorophyll pigment synthesis, chlorophyll fluorescence and photosynthetic rate of *Salix matsudana* seedlings reached their highest levels. In general, the optimal exogenous calcium concentration for alleviating salt stress and improving the photosynthetic characteristics of *Salix matsudana* seedlings was 10 mmol·L^-1^.

### 4.3 Exogenous calcium regulates the stress tolerance of *Salix matsudana* seedlings under NaCl stress

Salt deposition in older leaves of plants can cause premature ageing and inhibit photosynthesis and enzyme activity, and long-term reactions can lead to salt poisoning (Munns, 2005; De Oliveira et al., 2013; Roy et al., 2014). Gunes et al. (2007) found that plant cells are protected from oxidative damage by a series of antioxidant enzymes that remove or dispute the formation of reactive oxygen species. Rahman et al. (2016) also proved that SOD, POD and CAT are often thought of as crucial elements of the antioxidant defence of plants. In our experiments, the water use efficiency and SOD, CAT and POD activities of *Salix matsudana* seedlings gradually decreased with increasing NaCl concentrations. Ca^2+^ prevents cell membrane damage and leakage and stabilizes the cell membrane structure under adverse environmental conditions (Xu et al., 2017). Khan et al. (2012) found that antioxidative enzymes restore plant activity, leaves exposed to salt stress significantly increase antioxidative enzyme activity after SNP and CaCl_2_ are provided, leaves retain low electrolyte leakage, and H_2_O_2_ and TBARS levels are resistant to salt damage. Elkelish et al. (2019) also found that applying calcium could promote the synthesis of antioxidant and nonenzymatic antioxidant enzymes and increase their activity to repair the damage caused by salt stress. These results were consistent with the increase in SOD and POD activities when calcium was applied under salt stress (Xu et al., 2017). Adding Ca significantly increased salinity-mediated NR activity, suggesting that Ca effectively protects enzyme activity. Ca improves growth and photosynthesis by reducing Na intake (Elkelish et al., 2019). Jaleel et al. (2008) reported that the application of exogenous calcium could improve the injury of *Dioscorea rotundata* plants under salt stress and increase their resistance. The constant content of MDA is highly correlated with the high activities of POD and SOD, and adding calcium enables *Calligonum mongolicum* plants to resist oxidative damage under low and medium salt stress (Xu et al., 2017). Earlier results showed that cell membranes were affected by salt stress (Tuna et al., 2007). Ca^2+^ reduced oxidative damage by increasing antioxidant enzyme activity and regulating antioxidant defence and ROS detoxification systems (Roy et al., 2019). Our study showed that the antioxidant enzyme activity of *Salix matsudana* seedlings decreased with increasing NaCl concentration. Exogenous calcium could improve the CAT, SOD and POD activities of *Salix matsudana* seedlings under salt stress, showing a trend of first increasing and then decreasing. When the concentration of exogenous calcium was 10 mmol·L^-1^, the CAT, SOD and POD activities of *Salix matsudana* seedlings reached the highest. In conclusion, the optimal concentration of exogenous calcium for alleviating salt stress and improving the stress resistance of *Salix matsudana* seedlings was 10 mmol·L^-1^.

## 5 Conclusions

This study demonstrated that exogenous calcium could alleviate the physiological growth stress of *Salix matsudana* seedlings caused by snowmelt. The best exogenous calcium concentration was 10 mmol·L^-1^, which could promote the growth of *Salix matsudana* seedlings and increase their stomatal conductance, transpiration rate and net photosynthetic rate. The optimal exogenous calcium concentration could improve stress resistance, promote the accumulation of photosynthetic products and improve the water use efficiency of *Salix matsudana* seedlings. This study provided a feasible way to apply exogenous calcium to alleviate the harm of excessive snowmelt in northern winter to urban garden plants and promote plant growth.

## Data availability statement

The raw data supporting the conclusions of this article will be made available by the authors, without undue reservation.

## Author contributions

HL: conceptualization, methodology, writing - review and editing. SH: formal analysis, writing - original draft. CR: investigation. XW: resources, data curation SZ: writing - review and editing. LL: writing - review and editing JP: supervision, project administration. All authors contributed to the article and approved the submitted version.

## Funding

This research was supported by the Natural Science Foundation of China-Research (No. 3170030160, No. 41450007, No.31800364, No.31400611) and the Doctoral research start-up fund (No.880416020).

## Conflict of interest

The authors declare that the research was conducted in the absence of any commercial or financial relationships that could be construed as a potential conflict of interest.

## Publisher’s note

All claims expressed in this article are solely those of the authors and do not necessarily represent those of their affiliated organizations, or those of the publisher, the editors and the reviewers. Any product that may be evaluated in this article, or claim that may be made by its manufacturer, is not guaranteed or endorsed by the publisher.

## Abbreviations

Pn, Photosynthetic rate; Gs, Conductance to H_2_O; Tr, Transpiration rate; *F*_v_ */F*_0,_ the potential activity of PSII; *F*_v_ */F*_m,_ the maximum photochemical efficiency of PSII; WUE, Instantaneous water use efficiency; iWUE, Water use efficiency; SOD, Superoxide dismutase; CAT, Catalase; POD, Peroxidase.

## References

[B1] Acosta-Motos J. Ortuño M. Bernal-Vicente A. Diaz-Vivancos P. Sanchez-Blanco M. Hernandez J. (2017). Plant responses to salt stress: Adaptive mechanisms. Agronomy 7 (1), 18. doi: 10.3390/agronomy7010018

[B2] Aftab T. Khan M. M. A. Da Silva J. A. T. Idrees M. Naeem M. Moinuddin (2011). Role of salicylic acid in promoting salt stress tolerance and enhanced artemisinin production in *Artemisia annua* l. J. Plant Growth Regulation. 30 (4), 425–435. doi: 10.1007/s00344-011-9205-0

[B3] Alshammary S. F. Qian Y. L. Wallner S. J. (2004). Growth response of four turfgrass species to salinity. Agric. Water Manag. 66 (2), 97–111. doi: 10.1016/j.agwat.2003.11.002

[B4] Arnon D. I. (1949). Copper enzymes in isolated chloroplasts. polyphenoloxidase in *Beta vulgaris* . Plant Physiol. 24 (1), 1–15. doi: 10.1104/pp.24.1.1 16654194 PMC437905

[B5] Arshi A. Ahmad A. Aref I. M. Iqbal M. (2010). Calcium interaction with salinity-induced effects on growth and metabolism of soybean (*Glycine max* l.) cultivars. J. Environ. Biol. 31 (5), 795–801. doi: 10.1002/chin.200914162

[B6] Ashraf M. Harris P. J. C. (2004). Potential biochemical indicators of salinity tolerance in plants. Plant Sci. 166 (1), 3–16. doi: 10.1016/j.plantsci.2003.10.024

[B7] Bañón S. Miralles J. Ochoa J. Sánchez-Blanco M. J. (2012). The effect of salinity and high boron on growth, photosynthetic activity and mineral contents of two ornamental shrubs. Hortic. Sci. 39 (4), 188–194. doi: 10.17221/167/2011-hortsci

[B8] Blasius B. J. Merritt R. W. (2002). Field and laboratory investigations on the effects of road salt (NaCl) on stream macroinvertebrate communities. Environ. Pollution. 120 (2), 219–231. doi: 10.1016/s0269-7491(02)00142-2 12395833

[B9] Cabot C. Sibole J. V. Barceló J. Poschenrieder C. (2009). Sodium-calcium interactions with growth, water, and photosynthetic parameters in salt-treated beans. j. plant nutr. Soil Sci. 172 (5), 637–643. doi: 10.1002/jpln.200800124

[B10] Cha-um S. Singh H. P. Samphumphuang T. Kirdmanee C. (2012). Calcium-alleviated salt tolerance in indica rice (‘*Oryza sativa*’ l. spp. ‘Indica’): Physiological and morphological changes. Aust. J. Crop Sci. 6 (1), 176–182. doi: 10.3316/informit.054025225882532

[B11] Chen H. X. Li P. M. Gao H. Y. (2006). Alleviation of photoinhibition by calcium supplement in salt-treated *Rumex* leaves. Physiologia Plantarum. 129 (2), 386–396. doi: 10.1111/j.1399-3054.2006.00830.x

[B12] Dai H. L. Zhang K. L. Xu X. L. Yu H. Y. (2012). Evaluation on the effects of deicing chemicals on soil and water environment. Proc. Environ. Sci. 13, 2122–2130. doi: 10.1016/j.proenv.2012.01.201

[B13] Davis T. A. Volesky B. Mucci A. (2003). A review of the biochemistry of heavy metal biosorption by brown algae. Water Res. 37 (18), 4311–4330. doi: 10.1016/s0043-1354(03)00293-8 14511701

[B14] De Oliveira A. B. Mendes Alencar N. L. Gomes-Filho E. (2013). “Comparison between the water and salt stress effects on plant growth and development.” in Responses of organisms to water stress, vol. 4. Ed. Akıncı S. (London: IntechOpen Press), 67–94. doi: 10.5772/54223

[B15] Ding F. Chen M. Sui N. Wang B. S. (2010). Ca^2+^ significantly enhanced development and salt-secretion rate of salt glands of *Limonium bicolor* under NaCl treatment. South Afr. J. Botany. 76 (1), 95101. doi: 10.1016/j.sajb.2009.09.001

[B16] Doan C. D. Toshihiro M. Takeo Y. (2019). Effect of various drought stresses and subsequent recovery on proline, total soluble sugar and starch metabolisms in rice (*Oryza sativa* l.) varieties. Plant Product. Sci. 22 (4), 530–545. doi: 10.1080/1343943x.2019.1647787

[B17] Duarte B. Santos D. Marques J. C. Caçador I. (2013). Ecophysiological adaptations of two halophytes to salt stress: Photosynthesis, PS II photochemistry and anti-oxidant feedback– implications for resilience in climate change. Plant Physiol. Biochem. 67, 178–188. doi: 10.1016/j.plaphy.2013.03.004 23579080

[B18] Elkelish A. A. Alnusaire T. S. Soliman M. H. Gowayed S. Senousy H. H. Fahad S. (2019). Calcium availability regulates antioxidant system, physio-biochemical activities and alleviates salinity stress mediated oxidative damage in soybean seedlings. J. Appl. Bot. Food Qual. 92, 258–266. doi: 10.5073/JABFQ.2019.092.036

[B19] Ezzat A. Szab´o S. Szab´o Z. Heged˝us A. Ber´enyi D. Holb I. J. (2021). Temporal patterns and inter-correlations among physical and antioxidant attributes and enzyme activities of apricot fruit inoculated with monilinia iaxa under salicylic acid and methyl jasmonate treatments under shelf-life conditions. J. Fungi. 7 (5), 341. doi: 10.3390/jof7050341 PMC8145973 33925014

[B20] Fan X. H. Wu Y. H. Liu J. Y. (2013). Impacts of highway snow-melting agents on greening vegetation. Adv. Mat. Res. 831, 272–275. doi: 10.4028/www.scientific.net/amr.831.272

[B21] Fuller M. P. Hamza J. H. Rihan H. Z. Al-Issawi M. (2012). Germination of primed seed under NaCl stress in wheat. Int. Scholarly Res. Notices. 2012, 5. doi: 10.5402/2012/167804

[B22] García-Legaz M. F. López-Gómez E. Mataix-Beneyto J. Navarro A. Sánchez-Blanco M. J. (2008). Physiological behaviour of loquat and anger rootstocks in relation to salinity and calcium addition. J. Plant Physiol. 165 (10), 1049–1060. doi: 10.1016/j.jplph.2007.07.022 17997194

[B23] Gilliham M. Dayod M. Hocking B. J. Xu B. Conn S. J. Kaiser B. N. . (2011). Calcium delivery and storage in plant leaves: exploring the link with water flow. J. Exp. Botany. 62 (7), 2233–2250. doi: 10.1093/jxb/err111 21511913

[B24] Godwin K. S. Hafner S. D. Buff M. F. (2003). Long-term trends in sodium and chloride in the Mohawk river, new York: the effect of fifty years of road-salt application. Environ. Pollution. 124 (2), 273–281. doi: 10.1016/s0269-7491(02)00481-5 12713927

[B25] Gómez B. M. J. Álvarez S. Castillo M. Bañón S. Ortuño M. F. Sánchez B. ,. M. J. (2013). Water relations, nutrient content and developmental responses of euonymus plants irrigated with water of different degrees of salinity and quality. J. Plant Res. 126, 567–576. doi: 10.1007/s10265-012-0545-z 23306649

[B26] Goyal M. R. Gupta S. K. Singh A. (2018). “Physiological and biochemical changes in plants under soil salinity stress: a review,” in Engineering practices for management of soil salinity. Eds. Gupta S. K. Goyal M. R. Singh A. (New York: Apple Academic Press), 159–200.

[B27] Gunes A. Inal A. Alpaslan M. Eraslan F. Bagci E. G. Cicek N. (2007). Salicylic acid induced changes on some physiological parameters symptomatic for oxidative stress and mineral nutrition in maize (*Zea mays* l.) grown under salinity. J. Plant Physiol. 164 (6), 728–736. doi: 10.1016/j.jplph.2005.12.009 16690163

[B28] Guo Y. Liu Y. Zhang Y. Liu J. Gul Z. Guo X. R. . (2021). Effects of exogenous calcium on adaptive growth, photosynthesis, ion homeostasis and phenolics of *Gleditsia sinensis* lam. plants under salt stress. Agriculture 11 (10), 978. doi: 10.3390/agriculture11100978

[B29] Haiyun L. Kefu Z. Xiufeng W. (2002). The inhibition of salinity on the germination of halophyte seeds. J. Shandong Agric. Univ. 33 (2), 170–173.

[B30] Jaleel C. A. Gopi R. Gomathinayagam M. Panneerselvam R. (2008). Effects of calcium chloride on metabolism of salt-stressed dioscorea rotundata. Acta Biologica Cracoviensia Ser. Botanica. 50 (1), 63–67. doi: 10.1186/1471-2229-8-1

[B31] Jin J. Cui H. L. X. Yang Y. Wang Y. Lu X. (2016). Exogenous CaCl_2_ reduces salt stress in sour jujube by reducing na^+^ and increasing k^+^, Ca^2+^, and Mg^2+^ in different plant organs. J. Hortic. Sci. Biotechnol. 92 (1), 98–106. doi: 10.1080/14620316.2016.1228435

[B32] Kaya C. Kirnak H. Higgs D. Saltali K. (2002). Supplementary calcium enhances plant growth and fruit yield in strawberry cultivars grown at high (NaCl) salinity. Scientia Horticulturae. 93 (1), 65–74. doi: 10.1016/s0304-4238(01)00313-2

[B33] Kerstiens G. Tych W. Robinson M. F. Mansfield T. A. (2002). Sodium-related partial stomatal closure and salt tolerance of *Aster tripolium* . New Phytologist. 153 (3), 509–515. doi: 10.1046/j.0028-646x.2001.00330.x 33863213

[B34] Khan M. N. Siddiqui M. H. Mohammad F. Naeem M. (2012). Interactive role of nitric oxide and calcium chloride in enhancing tolerance to salt stress. Nitric. Oxide 27 (4), 210–218. doi: 10.1016/j.niox.2012.07.005 22884961

[B35] Khan M. N. Siddiqui M. H. Mohammad F. Naeem M. Khan M. M. A. (2009). Calcium chloride and gibberellic acid protect linseed (*Linum usitatissimum* l.) from NaCl stress by inducing antioxidative defence system and osmoprotectant accumulation. Acta Physiol. Plant 32 (1), 121–132. doi: 10.1007/s11738-009-0387-z

[B36] Kurth E. Cramer G. R. Lauchli A. Epstein E. (1986). Effects of NaCl and CaCl_2_ on cell enlargement and cell production in cotton roots. Plant Physiol. 82 (4), 1102–1106. doi: 10.2307/4270331 16665141 PMC1056265

[B37] Lee B. D. Choi Y. S. Kim Y. G. Kim I. S. Yang E. I. (2017). A comparison study of performance and environmental impacts of chloride-based deicers and eco-label certified deicers in south Korea. Cold Regions Sci. Technol. 143, 43–51. doi: 10.1016/j.coldregions.2017.08.010

[B38] Lei B. Huang Y. Xie J. J. Liu Z. X. Zhen A. Fan M. L. . (2013). Increased cucumber salt tolerance by grafting on pumpkin rootstock and after application of calcium. Biol. Plant 58, 179–184. doi: 10.1007/s10535-013-0349-6

[B39] Li H. Huo Y. Weng X. H. Zhou Y. B. Sun Y. Zhang G. Q. . (2022). Regulation of the growth of Mongolian pine (*Pinus sylvestris* var. *mongolica*) by calcium-water coupling in a semiarid region. Ecol. Indicators. 137, 108736. doi: 10.1016/j.ecolind.2022.108736

[B40] Liu Y. Xi M. Li Y. Cheng Z. Wang S. Kong F. (2021). Improvement in salt tolerance of *Iris pseudacorus* l. @ in constructed wetland by exogenous application of salicylic acid and calcium chloride. J. Environ. Manage. 300, 113703. doi: 10.1016/j.jenvman.2021.113703 34509818

[B41] Lu Q. H. Wang Y. Q. Yang H. B. (2021). Effect of exogenous calcium on physiological characteristics of salt tolerance in tartary buckwheat. Biologia 76 (12), 3621–3630. doi: 10.1007/s11756-021-00904-9

[B42] Maeda Y. Nakazawa R. (2008). Effects of the timing of calcium application on the alleviation of salt stress in the maize, tall fescue, and reed canary grass seedlings. Biol. Plant 52, 153–156. doi: 10.1007/s10535-008-0033-4

[B43] Mbarki S. Cerdà A. Zivcak M. Brestic M. Rabhi M. Mezni M. . (2018). Alfalfa crops amended with MSW compost can compensate the effect of salty water irrigation depending on the soil texture. Process Saf. Environ. Protection. 115, 8–16. doi: 10.1016/j.psep.2017.09.001

[B44] Miyazawa M. Pavan M. A. Block M. F. M. (1984). Determination of Ca, mg, K, Mn, Cu, zn, fe, and p in coffee, soybean, corn, sunflower, and pasture grass leaf tissues by a HCl extraction method. Commun. Soil Sci. Plant Anal. 15 (2), 141–147. doi: 10.1080/00103628409367462

[B45] Munns R. (2005). Genes and salt tolerance: bringing them together. New Phytologist. 167 (3), 645–663. doi: 10.1111/j.1469-8137.2005.01487.x 16101905

[B46] Murillo-Amador B. Jones H. G. Kaya C. Aguilar R. L. García-Hernández J. L. Troyo-Diéguez E. . (2006). Effects of foliar application of calcium nitrate on growth and physiological attributes of cowpea (*Vigna unguiculata* l. walp.) grown under salt stress. Environ. Exp. Botany. 58 (1-3), 188–196. doi: 10.1016/j.envexpbot.2005.08.003

[B47] Navarro J. M. Martıínez V. Carvajal M. (2000). Ammonium, bicarbonate and calcium effects on tomato plants grown under saline conditions. Plant Sci. 157 (1), 89–96. doi: 10.1016/s0168-9452(00)00272-7 10940472

[B48] Netondo G. W. Onyango J. C. Beck E. (2004). Sorghum and salinity: II. gas exchange and chlorophyll fluorescence of sorghum under salt stress. Crop Sci. 44 (3), 806–811. doi: 10.2135/cropsci2004.8060

[B49] Ozturk L. Demir Y. Unlukara A. Karatas I. Kurunc A. Duzdemir O. (2012). Effects of long-term salt stress on antioxidant system, chlorophyll and proline contents in pea leaves. Romanian Biotechnol. Letters. 17 (3), 7227–7236. doi: 10.1186/1754-6834-5-27

[B50] Paiva E. A. S. Sampaio R. A. Martinez H. E. P. (1998). Composition and quality of tomato fruit cultivated in nutrient solutions containing different calcium concentrations. J. Plant Nutr. 21 (12), 2653–2661. doi: 10.1080/01904169809365595

[B51] Parida A. Das A. B. Das P. (2002). NaCl Stress causes changes in photosynthetic pigments, proteins, and other metabolic components in the leaves of a true mangrove, *Bruguiera parviflora*, in hydroponic cultures. J. Plant Biol. 45, 28–36. doi: 10.1007/bf03030429

[B52] Parihar P. Singh S. Singh R. Singh V. P. Prasad S. M. (2014). Effect of salinity stress on plants and its tolerance strategies: a review. Environ. Sci. pollut. Res. 22, 4056–4075. doi: 10.1007/s11356-014-3739-1 25398215

[B53] Pathak J. Ahmed H. Kumari N. Pandey A. Rajneesh Sinha R. P. (2020). “Role of calcium and potassium in amelioration of environmental stress in plants,” in Protective chemical agents in the amelioration of plant abiotic stress: Biochemical and molecular perspectives. Eds. Aryadeep R. Durgesh K. T. (Hoboken: John Wiley and Sons Ltd Press), 535–562. doi: 10.1002/9781119552154.ch27

[B54] Pedersen L. B. Randrup T. B. Ingerslev M. (2000). Effects of road distance and protective measures on deicing NaCl deposition and soil solution chemistry in planted median strips. J. Arboriculture. 26 (5), 238–245. doi: 10.48044/jauf.2000.029

[B55] Perveen S. Saeed M. Parveen A. Javed M. T. Zafar S. Iqbal N. (2020). Modulation of growth and key physiobiochemical attributes after foliar application of zinc sulphate (ZnSO_4_) on wheat (*Triticum aestivum* l.) under cadmium (Cd) stress. Physiol. Mol. Biol. Plants. 26, 1787–1797. doi: 10.1007/s12298-020-00861-8 32943816 PMC7468032

[B56] Rahman A. Nahar K. Hasanuzzaman M. Fujita M. (2016). Calcium supplementation improves Na^+^/K^+^ ratio, antioxidant defense and glyoxalase systems in salt-stressed rice seedlings. Front. Plant Sci. 7. doi: 10.3389/fpls.2016.00609 PMC4864017 27242816

[B57] Rivett M. O. Cuthbert M. O. Gamble R. Connon L. E. Pearson A. Shepley M. G. . (2016). Highway deicing salt dynamic runoff to surface water and subsequent infiltration to groundwater during severe UK winters. Sci. Total Envi. 565, 324–338. doi: 10.1016/j.scitotenv.2016.04.09 27177139

[B58] Roy S. J. Negrão S. Tester M. (2014). Salt resistant crop plants. Curr. Opin. Biotechnol. 26, 115–124. doi: 10.1016/j.copbio.2013.12.004 24679267

[B59] Roy P. R. Tahjib-Ul-Arif M. Polash M. A. S. Hossen M. Z. Hossain M. A. (2019). Physiological mechanisms of exogenous calcium on alleviating salinity-induced stress in rice (*Oryza sativa* l.). Physiol. Mol. Biol. Plants. 25, 611–624. doi: 10.1007/s12298-019-00654-8 31168227 PMC6522628

[B60] Safdar H. Amin A. Shafiq Y. Ali A. Yasin R. Shoukat A. . (2019). A review: Impact of salinity on plant growth. Nat. Sci. 1, 34–40. doi: 10.7537/marsnsj170119.06

[B61] Schroeder J. I. Hagiwara S. (1989). Cytosolic calcium regulates ion channels in the plasma membrane of *Vicia faba* guard cells. Nature 338, 427–430. doi: 10.1038/338427a0

[B62] Song L. Zhu J. Yan Q. Li M. Yu G. (2015). Comparison of intrinsic water use efficiency between different aged *Pinus sylvestris* var. *mongolica* wide windbreaks in semiarid sandy land of northern China. Agroforest Syst. 89, 477–489. doi: 10.1007/s10457-014-9784-4

[B63] Stepien P. Johnson G. N. (2008). Contrasting responses of photosynthesis to salt stress in the glycophyte arabidopsis and the halophyte thellungiella: Role of the plastid terminal oxidase as an alternative electron sink. Plant Physiol. 149 (2), 1154–1165. doi: 10.1104/pp.108.132407 19052149 PMC2633845

[B64] Tahjib-Ul-Arif M. Roy P. R. Al-Mamun-Sohag A. Afrin S. Rady M. M. Hossain M. A. (2018). Exogenous calcium supplementation improves salinity tolerance in *BRRI Dhan28*; a salt-susceptible high-yielding *Oryza sativa* cultivar. J. Crop Sci. Biotechnol. 21, 383–394. doi: 10.1007/s12892-018-0098-0

[B65] Tang X. Mu X. Shao H. Wang H. Brestic M. (2014). Global plant-responding mechanisms to salt stress: physiological and molecular levels and implications in biotechnology. Crit. Rev. Biotechnol. 35 (4), 425–437. doi: 10.3109/07388551.2014.889080 24738851

[B66] Tao S. C. Yao J. L. Chen C. Y. Li L. Y. Kong Y. P. (2018). The impact of expressway snowmelt agent usage on the environment in an extreme freezing snow and sleet condition. IOP Conf. Ser.: Earth Environ. Sci. 191, 12073. doi: 10.1088/1755-1315/191/1/012073

[B67] Tian X. He M. Wang Z. Zhang J. Song Y. He Z. . (2015). Application of nitric oxide and calcium nitrate enhances tolerance of wheat seedlings to salt stress. Plant Growth Regul. 77, 343–356. doi: 10.1007/s10725-015-0069-3

[B68] Tuna A. L. Kaya C. Ashraf M. Altunlu H. Yokas I. Yagmur B. (2007). The effects of calcium sulphate on growth, membrane stability and nutrient uptake of tomato plants grown under salt stress. Environ. Exp. Botany. 59 (2), 173–178. doi: 10.1016/j.envexpbot.2005.12.007

[B69] Veselov D. S. Sharipova G. V. Veselov S. U. Kudoyarova G. R. (2008). The effects of NaCl treatment on water relations, growth, and ABA content in barley cultivars differing in drought tolerance. J. Plant Growth Regul. 27 (4), 380–386. doi: 10.1007/s00344-008-9064-5

[B70] Vysotskaya L. Hedley P. E. Sharipova G. Veselov D. Kudoyarova G. Morris J. . (2010). Effect of salinity on water relations of wild barley plants differing in salt tolerance. AoB PLANTS. 2010, 6. doi: 10.1093/aobpla/plq006 PMC3000697 22476064

[B71] Wang Y. Kang Y. Ma C. Miao R. Wu C. Long Y. . (2017). CNGC2 is a Ca^2+^ influx channel that prevents accumulation of apoplastic Ca^2+^ in the leaf. Plant Physiol. 173 (2), 1342–1354. doi: 10.1104/pp.16.01222 27999084 PMC5291024

[B72] Wang X. Lan Z. Tian L. Li J. Yang G. Gao Y. . (2021). Change of physiological properties and ion distribution by synergistic effect of Ca^2+^ and grafting under salt stress on cucumber seedlings. Agronomy 11 (5), 848. doi: 10.3390/AGRONOMY11050848

[B73] Weng X. Li H. Ren C. Zhou Y. Zhu W. Zhang S. . (2022). Calcium regulates growth and nutrient absorption in poplar seedlings. Front. Plant Sci. 13. doi: 10.3389/fpls.2022.887098 PMC9127976 35620692

[B74] Xue Y. F. Liu L. Liu Z. P. Mehta S. K. Zhao G. M. (2008). Protective role of Ca against NaCl toxicity in Jerusalem artichoke by up-regulation of antioxidant enzymes. Pedosphere 18 (6), 766–774. doi: 10.1016/s1002-0160(08)60072-7

[B75] Xu C. Li X. Zhang L. (2013). The effect of calcium chloride on growth, photosynthesis, and antioxidant responses of *Zoysia japonica* under drought conditions. PloS One 8 (7), e68214. doi: 10.1371/journal.pone.0068214 23844172 PMC3699550

[B76] Xu D. Wang W. Gao T. Fang X. Gao X. Li J. . (2017). Calcium alleviates decreases in photosynthesis under salt stress by enhancing antioxidant metabolism and adjusting solute accumulation in *Calligonum mongolicum* . Conserv. Physiol. 5 (1), cox060. doi: 10.1093/conphys/cox060

[B77] Yang W. Gao Y. Wang X. Li S. Zheng H. Chen Z. . (2022). Exogenous calcium application enhances salt tolerance of sweet sorghum seedlings. J. Agron. Crop Sci. 208 (4), 441–453. doi: 10.1111/jac.12585

[B78] Yang Y. Guo Y. (2017). Elucidating the molecular mechanisms mediating plant salt-stress responses. New Phytologist. 217 (2), 523–539. doi: 10.1111/nph.14920 29205383

[B79] Yang F. Li T. Zang Z. Lu S. (2010). Effects of timing of exogenous calcium application on the alleviation of salt stress in the tomato seedlings. Scientia Agricultura Sinica. 43 (6), 1181–1188. doi: 10.4028/www.scientific.net/AMM.37-38.1549

[B80] Yang S. Wang F. Guo F. Meng J. J. Li X. G. Wan S. B. (2014). Calcium contributes to photoprotection and repair of photosystem II in peanut leaves during heat and high irradiance. J. Integr. Plant Biol. 57 (5), 486–495. doi: 10.1111/jipb.12249 25103557

[B81] Yan C. Zhu H. Jin X. Yu L. (2017). Effect of deicing salt on seed germination of four main turfgrass species in North China. Grassland J. 25 (2), 437–441. doi: 10.11733/j.issn.1007-0435.2017.02.033

[B82] Yu S. W. Tang Z. C. (1998). Plant physiology and molecular biology (Beijing: Science Press).

[B83] Zhen-hua Z. Qiang L. Hai-xing S. Xiang-min R. Ismail A. M. (2012). Responses of different rice (*Oryza sativa* l.) genotypes to salt stress and relation to carbohydrate metabolism and chlorophyll content. Afr. J. Agric. Res. 7 (1), 19–27. doi: 10.5897/AJAR11.834

