# Supplementary figures and images for: Optimal exogenous calcium alleviates the damage of Snow-melting agent to Salix matsudana seedlings
Source: Front Plant Sci. 2022 Sep 28;13:928092. doi: 10.3389/fpls.2022.928092 (PMC9554415; doi:10.3389/fpls.2022.928092)

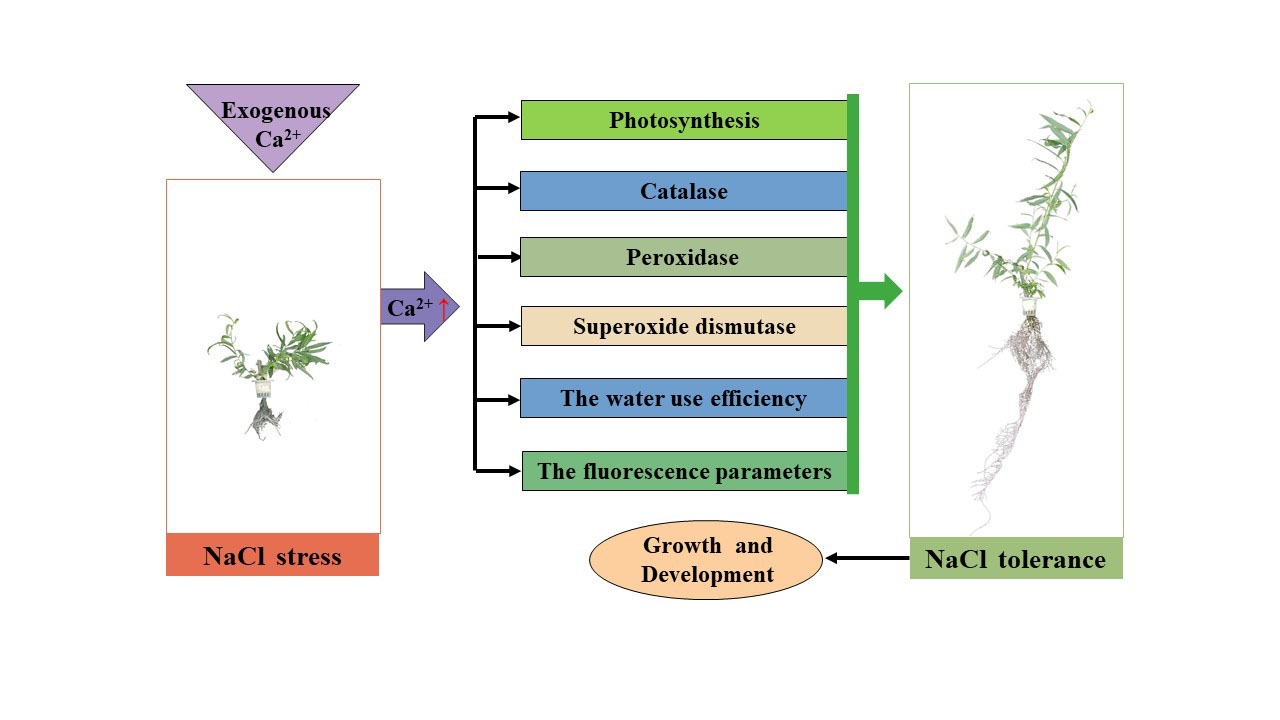

Supplement: Supplementary file 1 [file Image_1.jpg]
